# Altered amplitude of low‐frequency fluctuations and regional homogeneity in drug‐resistant epilepsy patients with vagal nerve stimulators under different current intensity

**DOI:** 10.1111/cns.13449

**Published:** 2020-09-23

**Authors:** Jin Zhu, Cuiping Xu, Xi Zhang, Liang Qiao, Xueyuan Wang, Xiaohua Zhang, Xiaoming Yan, Duanyu Ni, Tao Yu, Guojun Zhang, Yongjie Li

**Affiliations:** ^1^ Beijing Institute of Functional Neurosurgery Xuanwu Hospital Capital Medical University Beijing China

**Keywords:** drug‐resistant epilepsy, resting‐state functional mri, short‐term study, vagal nerve stimulation

## Abstract

**Background:**

The mechanisms of vagal nerve stimulation (VNS) for the treatment of drug‐resistant epilepsy (DRE) remain unclear. This study aimed to measure spontaneous brain activity changes caused by VNS in DRE patients using resting‐state functional MRI (rs‐fMRI).

**Methods:**

The rs‐fMRI scans were performed in 16 DRE patients who underwent VNS surgery. Amplitude of low‐frequency fluctuations (ALFF) and regional homogeneity (ReHo) was generated and examined using paired sample t‐test to compare activity changes at different current intensity stage. The preoperative and postoperative ALFF/ReHo were also compared in eight responders (≥50% reduction of seizure frequency three months after surgery) and eight nonresponders using paired sample t‐test.

**Results:**

The significant ALFF and ReHo changes were shown in various cortical/subcortical structures in patients under different current intensity. After three months of stimulation, responders exhibited increased ALFF in the right middle cingulate gyrus, left parahippocampal gyrus, and left cerebellum, and increased ReHo in the right postcentral gyrus, left precuneus, left postcentral gyrus, right superior parietal gyrus, right precentral gyrus, and right superior frontal gyrus. Nonresponders exhibited decreased ALFF in the left temporal lobe and right cerebellum, increased ALFF in bilateral brainstem, decreased ReHo in bilateral lingual gyri, and increased ReHo in the right middle frontal gyrus and right anterior cingulate gyrus.

**Conclusions:**

The spontaneous neural activity changes in DRE patients caused by VNS were in an ongoing process. Increased ALFF/ReHo in frontal cortex, cingulate gyri, precentral/postcentral gyri, parahippocampal gyri, precuneus, parietal cortex, and cerebellum may implicate in VNS‐induced improvement in seizure frequency.

## Graphical abstract

Dynamic amplitude of low‐frequency fluctuations and regional homogeneity changes existed in drug‐resistant epilepsy patients with vagal nerve stimulators (VNS) under different current intensity and may provide possible therapeutic mechanisms of VNS.

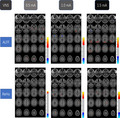

## 1 INTRODUCTION

Vagal nerve stimulation (VNS) was approved in 1997 by the US Food and Drug Administration for the treatment of drug‐resistant epilepsy (DRE) patients with partial seizures who are ≥12 years of age.^1^ Then, the age at implantation was extended to patients ≥4 years of age in 2017.^1^ In China, VNS was reported to be viable for DRE patients between 6 and 60 years of age.^2^ Since the first operation in 1988, more than 100 000 VNS stimulators have been implanted around the world.^3^ VNS has also been used for patients with various seizure types and epilepsy syndromes, including in children <4 years old.^4^

As an adjunctive therapy, numerous studies have reported that VNS can effectively reduce seizure frequency.^4^ After either two or three‐five years of stimulation, approximately 50% and 60% of epilepsy patients, respectively, were reported to achieve a ≥50% seizure reduction.^5^, ^6^ Commonly employed stimulation parameters are as follows: between 1.5 and 2.25 mA, 20‐30 Hz, 250‐500 μs, on time 30 s, off time 3‐5 min, which mainly based on doctors’ experience.^7^ It has been reported that the current intensity is an important factor of VNS outcome, and VNS can cause seizure reduction in a current range of 0.25‐2 mA.^8^, ^9^ A study on the relationship between clinical outcome and current intensity suggested that the high intensity of stimulation corresponds to a better outcome, although some other authors considered no significant correlation between them.^10^ In order to avoid laryngeal complications which started to appear when patients reached the output current of 1 mA, the device designed to produce electrical stimulation up to 3.5 mA was usually used at levels 1‐2 mA.^11^, ^12^, ^13^ Until now, owing to unclear therapeutic mechanisms, no surgical prediction criteria or parameter schedule has been proposed.

Resting‐state functional MRI (rs‐fMRI) was first proposed by Biswal to explore brain activity in 1995.^14^ The amplitude of low‐frequency fluctuation (ALFF) is a measure of rs‐fMRI which calculates the amplitude of each voxel in local brain regions in the frequency range of 0.01‐0.08 Hz.^15^ And the regional homogeneity (ReHo) is another measure to evaluate the similarity of the time series of a given voxel to those of its nearest neighbors in a voxel‐wise way based on Kendall's coefficient concordance (KCC).^16^ Both above two methods are commonly used in the rs‐fMRI study on spontaneous regional brain activity. In this study, we hypothesized that the increased current intensity participated in the process of seizure frequency reduction and selected ALFF and ReHo to analyze the changes of aberrant intrinsic brain activity in DRE patients with vagal nerve stimulators.

## 2 MATERIALS AND METHODS

### 2.1 Clinical data

16 DRE patients who underwent treatment with the VNS Therapy System (VNS Therapy, Cyberonics/LivaNova, Inc) in the Beijing Institute of Functional Neurosurgery between March and August 2019 were enrolled in this study. All patients were without other neurological disorders and previous surgical history, and underwent a detailed preoperative consultation in Beijing epilepsy consultation center. Considering the long‐term (interictal and ictal) video‐EEG, standard MRI, positron emission tomography, magnetoencephalography, and clinical manifestations, an optimal VNS surgery plan was worked out.

The seizure types, epilepsy types, and epilepsy syndromes were classified following the 2017 new International League Against Epilepsy Classification of the Epilepsies. The primary outcome measure was response rate defined using the equation: (seizures/month on VNS ‐ baseline seizures/month)/(baseline seizures/month) × 100%. The baseline period was three months before surgery and VNS period at every current intensity stage was one month. Responders were defined as those experiencing a seizure frequency reduction of ≥50% compared with the mean seizure frequency before implantation.

The VNS implantations were performed by two neurosurgeons according to standard procedures.^17^ Then, all patients followed an uniform VNS parameter schedule: Continuous electrical stimulation was commenced with 0.5 mA (current intensity), 30 Hz (signal frequency), 250 μs (pulse width), 30 s/5 minutes (on time/ off time) two days following the implantation; the current was gradually increased by 0.5 mA at standard intervals (every month) until it reached 1.5 mA intensity. So, when patients were followed up every month, the stimulation parameter was 0.5 mA, 30 Hz, 250 μs, 30 s/5 minutes at one month after surgery, 1.0 mA, 30 Hz, 250 μs, 30 s/ 5 minutes at two months after surgery, and 1.5 mA, 30 Hz, 250 μs, 30 s/ 5 minutes at three months after surgery. All subjects signed the informed consent.

### 2.2 Image processing and statistical analysis

The rs‐fMRI scans with a transmit/receive head coil were performed at one day before operation and one, two, three months after operation in 16 patients with DRE, respectively. There were no changes in the types or dosages of antiepileptic drugs during the time between preoperative and postoperative scan.

The process for the rs‐fMRI scan followed the VNS Therapy Manual (VNS Therapy Physicians’ Manual, LivaNova, Inc). The output current settings of the device were adjusted to 0 mA. The MRI examination was performed using a 3.0T scanner (Philips, Achieva TX). The BOLD fMRI sequence was single‐shot echo‐planar imaging with a 30‐ms echo time, 2000‐ms repetition time, 90°‐flip angel, 224 × 224‐mm field of view, and 64 × 64 image matrix. The scanning slice thickness and slice gaps were 3.5 mm and 0.5 mm, respectively. There was a total of 34 slices, with a scanning time of 8 min. The coronal images contained the AC‐PC line and were parallel to the corpus callosum. Patients were maintained in an absolutely quiet state during the scanning process. All 16 subjects completed the rs‐fMRI scan without discomfort, and the stimulators worked well when restarted.

The data format conversion and preprocessing were performed by DPARSF^18^ and included the following steps: (a) Data from the first 10 time points were removed to eliminate the nonuniform magnetic field and patients’ inadaptability to the environment, (b) slice timing correction, (c) head motion correction and removal of data with a parallel motion >1 mm and/or rotation >1, (d) time and spatial normalization, (e) registration of the BOLD image onto an individual T1 brain template, and (f) Gaussian smoothing (4 mm full width and half height), filtering (0.01 Hz < f < 0.08 Hz), and linear drift elimination.

We have extracted the mean ALFF and ReHo values, and tested the distribution of samples in SPSS 25.0 software. The results showed that all data accorded with normal distribution (P＞0.05) and were suitable for comparison by the paired sample *t*‐test. ALFF and ReHo were compared between the groups using a paired sample t‐test on a voxel basis (*P* < .001, GRF correction). The regions with altered values of the BOLD images were marked with red and yellow pixels when there were ≥10 continuous voxel collections (*P* < .001, GRF correction). Finally, the p values have been multiplied by 3 to compare the data between the baseline (0 mA) and 0.5 mA periods, between 0 mA and 1 mA periods, and 0 mA and 1.5 mA periods (Bonferroni correction). A *p* value < .05 was considered statistically significant.

## 3 RESULTS

### 3.1 Patients’ characteristics

There were ten males and six females (age range: 5‐41 years). According to the new International League Against Epilepsy classification, all cases were diagnosed as follows: seizure types (six focal, eight generalized, two unknown), epilepsy types (three focal, three generalized, ten combined focal and generalized), and epilepsy syndromes (one yes, 15 no). Six patients have MRI lesions (three patients with focal seizures and three patients with generalized seizures). The lateralization diagnosis was as follows: left in two patients with focal seizures, right in four patients with focal seizures, bilateral in eight patients with generalized seizures, unknown in two patients with unknown seizure types. A response rate ≥50% reduction in seizure frequency was observed in eight patients. The detailed information of antiepileptic drugs and above variables is listed in Table 1.

**Table 1:** Clinical data of 16 patients with drug‐resistant epilepsy

No.	Sex/Age (year)	Seizure types	Epilepsy types	Epilepsy syndromes	Antiepileptic drugs	MRI finding	Lateralization diagnosis	Responder
1	F/5	Focal	Generalized	No	Lamotrigine, oxcarbazepine, phenobarbital, phenytoin sodium, sodium valproate	Yes	Right	Yes
2	F/9	Unknown	Combined	No	Clonazepam, lamotrigine, sodium valproate, topiramate	No	Unknown	Yes
3	F/10	Focal	Combined	Dravet	Levetiracetam	Yes	Right	Yes
4	M/11	Generalized	Combined	No	Clonazepam, lacosamide, levetiracetam, sodium valproate, topiramate	No	Bilateral	Yes
5	M/19	Focal	Focal	No	Lamotrigine, oxcarbazepine, phenobarbital	Yes	Left	Yes
6	M/27	Generalized	Combined	No	Levetiracetam, oxcarbazepine, sodium valproate	Yes	Bilateral	Yes
7	M/28	Generalized	Generalized	No	Carbamazepine, sodium valproate	No	Bilateral	Yes
8	M/37	Focal	Focal	No	Oxcarbazepine	No	Right	Yes
9	F/5	Generalized	Combined	No	Levetiracetam, sodium valproate, topiramate	No	Bilateral	No
10	F/6	Generalized	Combined	No	Lamotrigine, levetiracetam, oxcarbazepine, topiramate	No	Bilateral	No
11	F/8	Generalized	Combined	No	Levetiracetam, oxcarbazepine	No	Bilateral	No
12	M/10	Generalized	Generalized	No	Clobazam, levetiracetam, sodium valproate	Yes	Bilateral	No
13	M/14	Focal	Combined	No	Clonazepam, lamotrigine, levetiracetam, quinidine, topiramate	No	Left	No
14	M/25	Focal	Focal	No	Carbamazepine, lamotrigine, levetiracetam, sodium valproate	No	Right	No
15	M/37	Unknown	Combined	No	Lamotrigine, levetiracetam	No	Unknown	No
16	M/41	Generalized	Combined	No	Oxcarbazepine, phenytoin sodium	Yes	Bilateral	No

Abbreviations: Combined, combined focal and generalized; F, female; M, male.

### 3.2 Changes of ALFF in 16 patients with different current intensity

Amplitude of low‐frequency fluctuations was generated from 16 patients with different VNS current intensity before and during 3 months after the operation (0, 0.5, 1.0, 1.5 mA). A paired sample t‐test was used to compare the ALFF data between groups.

**Figure 1:**
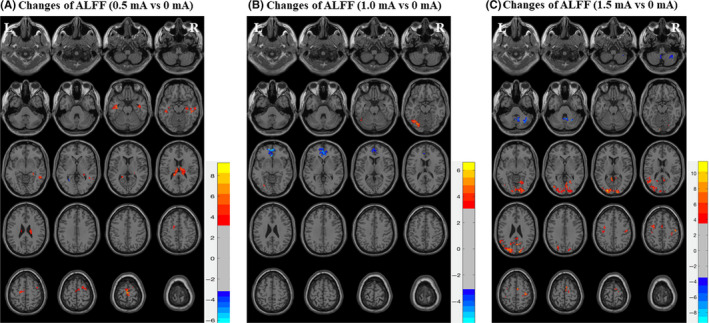
Changes of ALFF in 16 patients with different current intensity. The ALFF was generated from 16 patients with different VNS current intensity before and during three months after the operation (0, 0.5, 1.0, 1.5 mA). A paired sample *t*‐test was used to compare the ALFF data between groups. The brain regions with significant differences were displayed on standard brain templates (*P* < .001, GRF correction). A, Compared with preoperative values, the ALFF values increased in the right middle temporal gyrus, right hippocampus, right thalamus, right superior frontal gyrus, and left inferior temporal gyrus in patients with 0.5 mA. B, The ALFF decreased in bilateral anterior cingulate gyrus, but increased in left fusiform gyrus in patients with 1.0 mA. C, The ALFF decreased in the bilateral cerebellum, but increased in the left middle occipital gyrus, left lingual gyrus, left superior occipital gyrus, left middle temporal gyrus, bilateral precentral gyrus, and left postcentral gyrus in patients with 1.5 mA

The brain regions with significant differences were displayed on standard brain templates (*P* < .001, GRF correction) (Figure 1A‐C). Compared with preoperative values, the ALFF values increased in the right middle temporal gyrus, right hippocampus, right thalamus, right superior frontal gyrus, and left inferior temporal gyrus in patients with 0.5 mA; the ALFF decreased in bilateral anterior cingulate gyrus, but increased in left fusiform gyrus in patients with 1.0 mA; the ALFF decreased in the bilateral cerebellum, but increased in the left middle occipital gyrus, left lingual gyrus, left superior occipital gyrus, left middle temporal gyrus, bilateral precentral gyrus, and left postcentral gyrus in patients with 1.5 mA (Table 2).

**Table 2:** Brain areas with significantly different ALFF in 16 patients with different current intensity (p t‐test, Bonferroni correction)

Groups	Brain areas	Voxels	MNI coordinates			*t* value	*p* value
			*X*	*Y*	*Z*		
0.5 vs 0 (mA)	Middle temporal gyrus (R)	50	55	−39	−9	5.8071	<.05
	Hippocampus (R)	31	31	−17	−17	3.8908	<.05
	Thalamus (R)	37	13	−17	15	4.1938	<.05
	Superior frontal gyrus (R)	44	22	2	60	4.0965	<.05
	Inferior temporal gyrus (L)	23	−41	−14	−24	4.4971	<.05
1.0 vs 0 (mA)	Anterior cingulate gyrus (L)	98	−6	44	3	−3.303	<.05
	Anterior cingulate gyrus (R)	26	1	42	3	−4.195	<.05
	Fusiform gyrus (L)	70	−37	−67	−14	3.749	<.05
1.5 vs 0 (mA)	Cerebellum (R)	130	36	−63	−42	−8.3555	<.05
	Cerebellum (L)	72	−6	−48	−45	−9.5888	<.05
	Middle occipital gyrus (L)	197	−19	−93	7	4.2273	<.05
	Lingual gyrus (L)	165	0	−3	44	4.3246	<.05
	Superior occipital gyrus (L)	83	−25	−84	23	6.7248	<.05
	Middle temporal gyrus (L)	43	−47	−56	19	4.228	<.05
	Precentral gyrus (R)	44	34	−25	58	5.8521	<.05
	Precentral gyrus (L)	50	−33	−4	45	8.2267	<.05
	Postcentral gyrus (L)	40	−40	−29	50	3.5229	<.05

Abbreviations: ALFF, amplitude of low‐frequency fluctuation; L, left; MNI, Montreal Neurological Institute; R, right.

### 3.3 Changes of ReHo in 16 patients with different current intensity

ReHo was generated from 16 patients with different VNS current intensity before and during 3 months after the operation (0, 0.5, 1.0, 1.5 mA). A comparison of ReHo data before and after the operation was performed using a paired sample t‐test.

**Figure 2:**
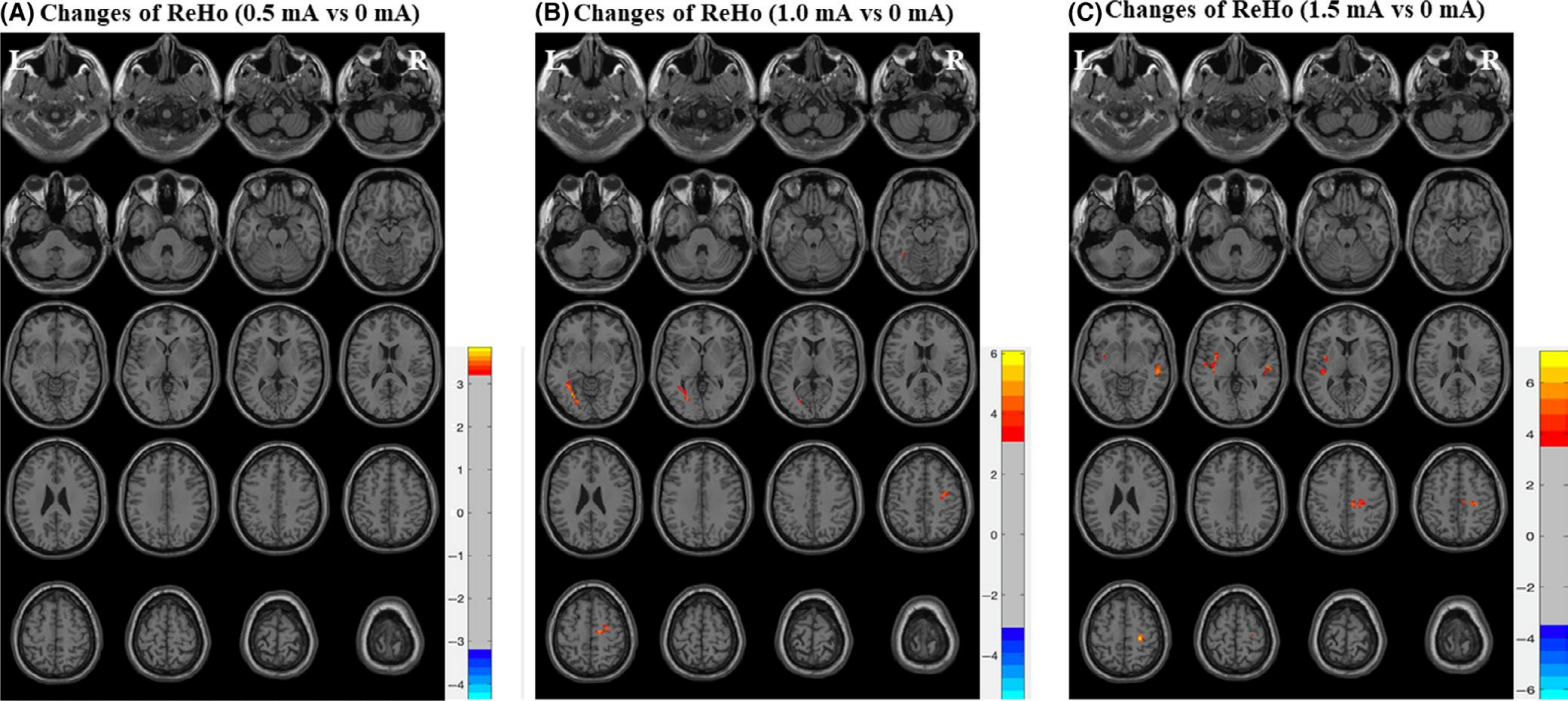
Changes of ReHo in 16 patients with different current intensity. The ReHo was generated from 16 patients with different VNS current intensity before and during three months after the operation (0, 0.5, 1.0, 1.5 mA). A paired sample *t*‐test was used to compare the ReHo data between groups. The brain regions with significant differences were displayed on standard brain templates (*P* < .001, GRF correction). A, Compared with the preoperative state, no regions showed increased or decreased ReHo in patients with 0.5 mA. B, ReHo increased in the right precentral gyrus and left fusiform gyrus in patients with 1.0 mA. C, ReHo increased in right middle temporal gyrus, left superior temporal gyrus, right precentral gyrus, and right middle cingulate gyrus in patients with 1.5 mA

The brain regions with significant differences were displayed on standard brain templates (*P* < .001, GRF correction) (Figure 2A‐C). Compared with the preoperative state, no regions showed increased or decreased ReHo in patients with 0.5 mA; ReHo increased in the right precentral gyrus and left fusiform gyrus in patients with 1.0 mA; ReHo increased in right middle temporal gyrus, left superior temporal gyrus, right precentral gyrus, and right middle cingulate gyrus in patients with 1.5 mA (Table 3).

**Table 3:** Brain areas with significantly different ReHo in 16 patients with different current intensity (p t‐test, Bonferroni correction)

Groups	Brain areas	Voxels	MNI coordinates			*t* value	*p* value
			*X*	*Y*	*Z*		
0.5 vs 0 (mA)	None						
1.0 vs 0 (mA)	Precentral gyrus (R)	25	33	−11	50	4.5516	<.05
	Fusiform gyrus (L)	37	−33	−54	−12	4.5465	<.05
1.5 vs 0 (mA)	Middle temporal gyrus (R)	40	55	−12	−4	4.2225	<.05
	Superior temporal gyrus (L)	40	−41	−14	−2	4.7838	<.05
	Precentral gyrus (R)	20	27	−22	57	5.2824	<.05
	Middle cingulate gyrus (R)	23	16	−26	44	4.6637	<.05

Abbreviations: L, left; MNI, Montreal Neurological Institute; R, right; ReHo, regional homogeneity.

### 3.4 Changes in ALFF before and after the operation in responders and nonresponders

Amplitude of low‐frequency fluctuations was generated from 16 patients with a VNS stimulator who underwent an rs‐fMRI scan before and at three months after the operation. Patients were divided into two groups: responders (n = 8) and nonresponders (n = 8). The ALFF generated from each group was analyzed before and after the operation. A paired sample t‐test was used to compare the ALFF data before and after the operation.

**Figure 3:**
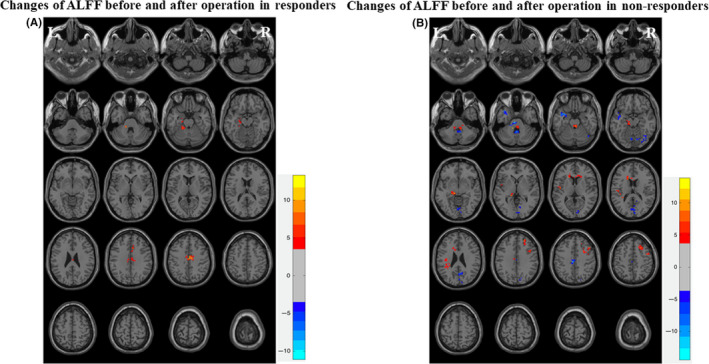
Changes of ALFF before and after operation in responders and nonresponders. A, In responders after three months of stimulation, the ALFF values increased in right middle cingulate gyrus, left parahippocampal gyrus, and left cerebellum in responders using the standard brain templates (*P* < .001, GRF correction). B, In nonresponders after three months of stimulation, ALFF decreased in the left temporal lobe and right cerebellum, and increased in the bilateral brainstem in nonresponders using the standard brain templates (*P* < .001, GRF correction)

There was significant difference in ALFF in various brain regions using the standard brain templates (*P* < .001, GRF correction). After three months of stimulation, the ALFF values increased in the right middle cingulate gyrus, left parahippocampal gyrus, and left cerebellum in responders (Figure 3A), while ALFF decreased in the left temporal lobe and right cerebellum, and increased in the bilateral brainstem in nonresponders (Figure 3B). The brain areas with significantly different ALFF values before and after the operation are listed in Table 4.

**Table 4:** Brain areas with significantly different ALFF values before and after surgery (p t‐test, Bonferroni correction)

Groups	Brain areas	Voxels	MNI coordinates			*t* value	*p* value
			*x*	*y*	*z*		
Responders	AO > BO						
	Middle cingulate gyrus (R)	132	3	−9	42	11.579	<.05
	Parahippocampal gyrus (L)	40	−21	−21	−18	8.469	<.05
	Cerebellum (L)	19	−18	−40	−25	5.996	<.05
Nonresponders	AO < BO						
	Temporal lobe (L)	42	−30	3	−24	−11.11	<.05
	Cerebellum (R)	18	0	−51	−30	−9.665	<.05
	AO > BO						
	Brainstem (L)	29	−12	−24	−12	5.702	<.05
	Brainstem (R)	27	3	−33	−24	8.967	<.05

Abbreviations: ALFF, amplitude of low‐frequency fluctuation; AO, after operation; BO, before operation; L, left; MNI, Montreal Neurological Institute; R, right.

### 3.5 Changes in ReHo before and after the operation in responders and nonresponders

**Figure 4:**
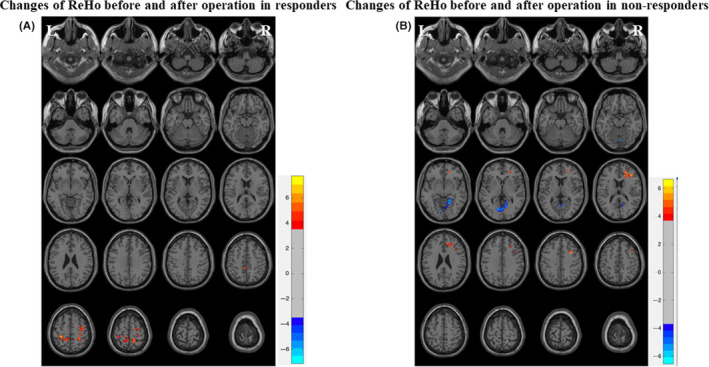
Changes of ReHo before and after operation in responders and nonresponders. A, In responders after three months of stimulation, ReHo increased in the right postcentral gyrus, left precuneus, left postcentral gyrus, right superior parietal gyrus, right precentral gyrus, and right superior frontal gyrus in responders using the standard brain templates (*P* < .001, GRF correction). B, In nonresponders after three months of stimulation, ReHo decreased in the bilateral lingual gyrus, and increased in the right middle frontal gyrus and right anterior cingulate gyrus in nonresponders (*P* < .001, GRF correction)

ReHo was generated from 16 patients with a VNS stimulator who underwent an rs‐fMRI scan before and at 3 months after the operation. A comparison of ReHo data before and after the operation was performed on each group with imaging data using a paired sample *t*‐test (*P* < .001, GRF correction). The brain regions with significant differences were displayed on standard brain templates. Compared with the preoperative state, ReHo increased in the right postcentral gyrus, left precuneus, left postcentral gyrus, right superior parietal gyrus, right precentral gyrus, and right superior frontal gyrus in responders (Figure 4A), while ReHo decreased in the bilateral lingual gyrus and increased in the right middle frontal gyrus and right anterior cingulate gyrus in nonresponders (Figure 4B). The brain areas with significantly different ReHo before and after the operation are listed in Table 5.

**Table 5:** Brain areas with significantly different ReHo before and after surgery (p t‐test, Bonferroni correction)

Groups	Brain areas	Voxels	MNI coordinates			*t* value	*p* value
			*x*	*y*	*z*		
Responders	AO > BO						
	Postcentral gyrus (R)	63	27	−42	54	7.221	<.05
	Precuneus (L)	47	−17	−41	58	3.967	<.05
	Postcentral gyrus (L)	45	−27	−37	57	5.736	<.05
	Superior parietal gyrus (R)	31	16	−48	65	5.121	<.05
	Precentral gyrus (R)	30	36	−9	60	7.75	<.05
	Superior frontal gyrus (R)	24	18	−11	63	3.643	<.05
Nonresponders	AO < BO						
	Lingual gyrus (R)	91	12	−64	1	−5.242	<.05
	Lingual gyrus (L)	48	−6	−74	1	−4.71	<.05
	AO > BO						
	Middle frontal gyrus (R)	69	24	42	15	5.69	<.05
	Anterior cingulate gyrus (R)	14	13	35	23	5.421	<.05

Abbreviations: AO, after operation; BO, before operation; L, left; MNI, Montreal Neurological Institute; R, right; ReHo, regional homogeneity.

## 4 DISCUSSION

DRE was defined as that more than four seizures per month occurred after more than two years of regular antiepileptic drugs and reaching the maximum dose that patients can tolerate.^19^ Despite rapid advances in effective drug treatments and surgical techniques, 15%‐40% of epilepsy patients remain unable to effectively control seizures.^20^ Neuromodulation is an optimal treatment for patients for whom resection surgery is unsuitable and can effectively reduce the frequency and severity of seizures.^21^ With more than 20 years of development, VNS has become the most widely used operation for patients with DRE.^22^ More than 50% of patients can achieve ≥50% seizure reduction with a VNS stimulator, with few operative complications.^23^ However, it is not possible to predict the outcome of the operation, and there are no criteria for selecting appropriate patients, largely because the mechanism of action of VNS remains unclear.

Numerous studies have examined the changes in the brain after VNS. The vagal‐locus coeruleus‐hippocampal noradrenergic pathway is thought to represent a key mechanism in VNS.^24^ For example, VNS was reported to cause significant changes in blood flow in the thalamus, hypothalamus, hippocampus, and amygdala.^25^ VNS may play an antiepileptic role by altering synaptic activity in the thalamus.^26^ Imaging studies have also shown that VNS can cause changes in activity in the insula, amygdala, hippocampus, parahippocampus, thalamus, and cerebellum.^27^ Further, thalamic activation measured by BOLD fMRI was associated with improved VNS treatment response in patients with seizures.^28^ Thalamic connections to the anterior cingulate and insular cortices are stronger in VNS responders.^29^ Further, the default mode network, salience network, and ascending reticular activating system were reported to have close associations with the generation and propagation of epileptic activity.^30^, ^31^

The ALFF and ReHo provide useful tools in rs‐fMRI for the study of epilepsy.^32^ In the present study, we defined the responder rate as ≥50% seizure frequency improvement from baseline.^33^ There were no changes in the types or dosages of antiepileptic drugs during the time between two rs‐fMRI scans. ALFF and ReHo were chosen as methods to analyze the characteristics of slow wave oscillations and functional activity in local brain regions.

This rs‐fMRI study was made to explore aberrant intrinsic brain activity in epilepsy patients with different current intensity. Our important findings were that there were changes in the ALFF and ReHo with the enhanced current intensity. Specifically, the ALFF values increased in the right middle temporal gyrus, right hippocampus, right thalamus, right superior frontal gyrus, and left inferior temporal gyrus (0.5 mA); the ALFF decreased in bilateral anterior cingulate gyrus, but increased in left fusiform gyrus (1.0 mA); the ALFF decreased in the bilateral cerebellum, but increased in the left middle occipital gyrus, left lingual gyrus, left superior occipital gyrus, left middle temporal gyrus, bilateral precentral gyrus, and left postcentral gyrus (1.5 mA). ReHo increased in the right precentral gyrus and left fusiform gyrus (1.0 mA); ReHo increased in right middle temporal gyrus, left superior temporal gyrus, right precentral gyrus, and right middle cingulate gyrus (1.5 mA).

To date, relatively few studies investigating the effects of various VNS parameters in vivo have been conducted. As we know, this is the first longitudinal resting‐state fMRI study to explore aberrant intrinsic brain activity in epilepsy patients with different current intensity of VNS. In order to avoid interference from stimulation time and other stimulation parameters, only current intensity was changed during the three months of stimulation, and a paired sample t‐test was chosen for analysis of ALFF and ReHo before and after operation. The above results may provide meaningful evidence for that short‐term stimulations at different current intensities cause spontaneous neural activity changes in some brain regions in DRE patients. The brain functional reorganization suggests a possible mechanism of VNS.

Our other key findings were that at 3 months after stimulation, ALFF increased in right middle cingulate gyrus, left parahippocampal gyrus, and left cerebellum in responders, and decreased in the left temporal lobe and right cerebellum, and increased in the bilateral brainstem in nonresponders. Further, ReHo increased in the right superior frontal gyrus, left precuneus, bilateral postcentral gyri, right precentral gyrus, and right superior parietal gyrus, in responders; and decreased in the bilateral lingual gyrus, and increased in the right middle frontal gyrus and right anterior cingulate gyrus in nonresponders. It is reported that the vagal afferent network (VAN) which mainly transmit somatosensory and visceral sensory signals from the bronchi, lungs, heart, and esophagus, to the brainstem, subcortical, and cortical nuclei. The observation in this study indicates that the cortical and subcortical structures with functional activity changes are important for VNS. Increased activity in the precentral/postcentral gyri, cingulate gyri, cerebellum, which is associated with motor execution and coordination, may also provide the available evidence for the use of neurostimulation to treat DRE, including transcranial magnetic stimulation (precentral gyrus as the target), cingulate cortex stimulation (cingulate cortex as the target), and cerebellar stimulation (cerebellum as the target).^34^, ^35^, ^36^ In addition, our findings also support that 3.0T MRI is safe and beneficial for follow‐up of epilepsy patients with VNS.^37^

This is the first rs‐fMRI study on spontaneous neural activity changes in DRE patients with different current intensity of VNS, and the findings provide possible mechanisms of VNS. However, epilepsy is regarded as a disruption of functional networks, and its pathogenesis is very complex.^27^ Many critical structures may participate in the seizure control of VNS.

Because doctors often need to increase the current intensity (0.25 mA/ titration) or adjust other stimulus parameters according to the clinical symptom improvement of patients after reaching 1.5 mA, it is difficult to unify the stimulus parameters of patients at the same time interval. We analyzed the fMRI data from relatively small samples under four current intensities (0, 0.5, 1.0 and 1.5 mA) on the basis of normal diagnosis and treatment work, and the follow‐up time in this study was relatively short to restrict changes of parameters and antiepileptic drugs. The limitations of the present study are as follows; relatively small case number, short stimulation period, and no analysis of epileptic network connections. Additionally, there was no wash‐out period before new stimulation period as these patients have frequent seizures. It is worth to make future study with larger number of cases and longer VNS duration to explore the activity changes of brain networks caused by VNS in epilepsy patients.

## CONFLICT OF INTEREST

The authors declare no financial or commercial conflict of interest.

## ETHICAL APPROVAL

All procedures performed in the studies involving human participants were in accordance with the ethical standards of the institutional and/or national research committee and with the 1964 Helsinki Declaration and its later amendments or comparable ethical standards.
